# Food Deprivation in *Triatoma pallidipennis* Increases the Expression of α-Tubulin, β-Actin, and a Heat Shock Protein in the Anterior Midgut

**DOI:** 10.3390/pathogens15050482

**Published:** 2026-04-30

**Authors:** Olivia Alicia Reynoso-Ducoing, Elsa Gabriela Díaz-Ramírez, Elia Torres-Gutiérrez, Mauro Omar Vences-Blanco, Berenice González-Rete, Yolanda Guevara-Gómez, Margarita Cabrera-Bravo, Paz María Silvia Salazar-Schettino

**Affiliations:** Departamento de Microbiología y Parasitología, Facultad de Medicina, Universidad Nacional Autónoma de México, México City 04510, Mexico; oard@unam.mx (O.A.R.-D.);

**Keywords:** fed insects, unfed insects, protein expression, β-actin, α-tubulin, HSP70, anterior midgut, rectum, *Triatoma pallidipennis*

## Abstract

Food deprivation induces intestinal adaptations in *Triatoma pallidipennis*, a hematophagous insect with intermittent feeding habits. The ability to survive long periods without food promotes the persistence of this vector in the environment and contributes to its evolutionary success. This study employed one- and two-dimensional electrophoretic techniques combined with Western blot to evaluate the abundance of α-tubulin, β-actin, and the heat shock protein HSP70. These proteins were more abundant in the anterior midgut tissue of unfed insects than in that of fed insects. As these responses were similar in females and males, the observed adaptations primarily depend on feeding status and intestinal region. These findings provide further insight into the intestinal physiology of *T. pallidipennis*, a vector of the flagellate parasite *Trypanosoma cruzi*, the causative agent of Chagas disease.

## 1. Introduction

Chagas disease, also known as American trypanosomiasis [1], is a parasitic infection caused by the flagellate protozoan *Trypanosoma cruzi*. Its endemic region extends from the southern United States to southern Argentina and Chile [2], representing a significant public health concern that is further exacerbated by social, ecological, and housing factors. Among the insect vectors involved in its transmission, *Triatoma pallidipennis* (Stal, 1872) (Hemiptera: Reduviidae), also known as *Meccus pallidipennis*, is a hematophagous species widely distributed across thirteen Mexican states [1], where it is considered one of the three principal vectors of *T. cruzi* [3,4]. This species inhabits a broad range of ecological niches and maintains close contact with human populations, contributing to transmission.

Food availability is a key factor influencing the insect life cycle, as it is associated with physiological and behavioral responses such as increased host-seeking activity and oviposition. However, there is still limited information on the proteins that mediate these processes at the cellular level in triatomines. Advances in proteomic approaches, particularly two-dimensional electrophoresis, have enabled the characterization of protein abundance, isoforms, and functional states across intestinal tissues and physiological conditions [5].

The digestive system of *T. pallidipennis* consists of three main tissues. (1) The anterior midgut (AMG), also referred to as the promesenteron, stomach, gut, or crop, functions in the storage, concentration, and initial processing of ingested blood. This region has a near-neutral pH and contributes to water removal from the food bolus as part of the early stages of digestion [6]. Erythrocyte lysis and intracellular lipid storage also occur in the AMG [7]. (2) The posterior midgut, also known as the postmesenteron or small intestine, produces the perimicrovillar membrane after blood ingestion, facilitating digestion and nutrient absorption. (3) The final region is the rectum (RE), also referred to as the proctodeum or hindgut, which is involved in nutrient absorption and waste excretion [8,9].

The cytoskeleton of insect intestinal cells is composed primarily of actin microfilaments and microtubules. Actin supports the structure of microvilli and the cortical cytoskeleton of intestinal epithelial cells and is involved in adhesion, morphological changes, and endo- and exocytosis through interactions with myosin and regulatory proteins such as Arp2/3 and cofilin. Microtubules regulate intracellular transport, including the delivery and recycling of vesicles and organelles, and are essential for mitotic spindle formation [10]. In insects, intermediate filaments are largely absent; instead, nuclear lamins (type-V filaments) form the nuclear lamina [11].

Actin and tubulin are essential for insect gut morphophysiology. These proteins provide structural support to the digestive epithelium and facilitate the transport of molecules required for digestion. Actin, located in the microvilli, regulates the trafficking of digestive enzymes to the cell surface, promoting nutrient absorption and enabling digestion at the perimicrovillar membrane [12,13,14]. Tubulin participates in the vesicular transport of enzymes, membrane proteins, and nutrients, playing a central role in intestinal cellular function [15,16]. The heat shock protein HSP70 is involved in maintaining protein stability, preventing misfolding, and protecting intestinal cells under stress conditions. They also contribute to metabolic regulation and immune responses associated with feeding [17,18].

The AMG epithelium is characterized by the presence of microvilli with a central core of F-actin. Beneath this structure lies a subapical actin-rich network that anchors microvilli, connects to lateral junctions, and serves as a platform for membrane trafficking [19]. Actin provides structural support and barrier function, while microtubules facilitate polarized vesicle transport, including the delivery and recycling of membrane components and digestive enzymes [20].

The RE of triatomines exhibits a specialized, cuticularized epithelium adapted for ion and water reabsorption [21,22]. This barrier relies on cortical actin, which interacts with septate junctions to provide mechanical resistance to large volume changes and osmotic stress [9,23,24,25]. Microtubules function as intracellular tracks that direct the traffic and recycling of ion pumps and channels, enabling rapid physiological adjustments.

HSP70 has been shown to protect the midgut of *Aedes aegypti* from thermal stress associated with blood ingestion [10,26]. Its expression peaks within the first hour after ingestion and decreases gradually over the next 24 h. Similar responses have been observed in other hematophagous insects, including *Culex pipiens*, *Anopheles gambiae*, and *Cimex lectularius*. Therefore, HSP70-mediated protection seems to be a conserved mechanism among hematophagous arthropods [27].

Several studies have identified proteins and transcripts in the intestine of *T. pallidipennis* [6,8,9,27]. However, only a limited number of reports have focused specifically on glycoproteins [28,29,30,31], highlighting the lack of comprehensive information on protein abundance and distribution in triatomine guts.

Western blot analysis has been widely used to assess protein abundance based on densitometric quantification of immunoreactive signals. These measurements rely on specialized software to provide accurate and reproducible comparisons between experimental conditions [32,33,34,35,36,37].

In this study, one- and two-dimensional electrophoresis and Western blotting were used to analyze cytoskeletal proteins and HSP70 in unfed and fed *T. pallidipennis*. Specifically, the abundance of β-actin, α-tubulin, and HSP70 was evaluated in the AMG and RE tissues of female and male insects under different feeding conditions. These findings contribute to a better understanding of insect digestive physiology and may support the identification of potential molecular targets for vector control strategies against *T. cruzi* transmission.

## 2. Materials and Methods

### 2.1. Insect Provenance and Experimental Groups

*T. pallidipennis* specimens were obtained from the insectarium of the Parasite Biology Laboratory in the Department of Microbiology and Parasitology at the Faculty of Medicine, UNAM.

Two groups of adult insects were randomly established. Each group consisted of 15 females and 15 males. The insects in the first group were kept unfed for 21 days after molting into adulthood and were then dissected. Insects in the fed group ingested blood from CD-1 mice eight days after molting into adults and were dissected fifteen days after feeding. The use of animals was approved by the Internal Committee for the Care and Use of Laboratory Animals (CICUAL 013-CIC-2022, Faculty of Medicine, UNAM, Ciudad de México, Mexico).

### 2.2. Intestine Dissection and Recovery of AMG and RE

Insects were dissected under a stereoscopic microscope (Stemi 2000, Carl Zeiss, Jena, Germany). Legs were removed with dissecting forceps and the insect was placed in a Petri dish at 4 °C. The abdomen was disinfected with 70% ethanol. The abdomen was opened via the connexiva [38]. The intestine was dissected, and the AMG and RE were separated. Tissues were washed thoroughly with phosphate buffer solution to reduce contamination with intestinal contents (40 mM KCl, 1 mM MgCl_2_, and 6.7 mM phosphate buffer, pH 7.4), supplemented with a 4X protease inhibitor cocktail (Complete, Thermo Scientific, Waltham, MA, USA), 0.1 mM phenylmethylsulfonyl fluoride (PMSF, Sigma, Burlington, MA, USA), and 1 µM pepstatin A (Sigma), all at pH 7.4 and 4 °C [39].

Tissues of 15 individuals from the same intestinal region, group, and sex were pooled, possessing average wet weights of 0.9664 g for AMG and 0.3650 g for RE. Tissues from the four experimental groups were frozen at −80 °C in phosphate buffer until protein extraction.

### 2.3. Protein Extraction, Purification, Concentration, and Quantification

Pools were thawed and processed independently. A modified version of a previously reported protein extraction protocol was used [40]. Tissues were lysed in a phosphate buffer containing protease inhibitors by sonication in three 20-second cycles at 30% amplitude with one-minute intervals at rest in a Sonifier SFX150 ultrasonic processor (Branson Ultrasonics, Brookfield, CT, USA). Proteins were precipitated with 10% trichloroacetic acid and 20 mM 1,4-bis(sulfanyl)butane-2,3-diol (dithiothreitol) in cold acetone and solubilized in phosphate buffer pH 7.4 with protease inhibitors [41].

Protein quantification was performed using the Pierce BCA Protein Assay Kit and 2 mg/mL bovine serum albumin as standard (Thermo Scientific). These proteins were stored at −80 °C until electrophoretic analysis.

### 2.4. One-Dimensional Electrophoresis

Protein extracts were prepared according to the manufacturer’s instructions for the Invitrogen NuPAGE™ system (Invitrogen, Thermo Fisher Scientific, Waltham, MA, USA). Each sample was mixed with 1 µL of Sample Reducing Agent and 2.5 µL of 4× lithium dodecyl sulfate Sample Buffer and adjusted to a final volume of 10 µL with Milli-Q water, corresponding to 20 µg of protein per lane. When the initial sample volume exceeded 10 µL, reagent volumes were scaled proportionally to maintain the recommended reagent-to-sample ratios. Electrophoresis was performed under reducing conditions using a NuPAGE™ system in a Mini Gel Tank (Invitrogen). Seven-centimeter NuPAGE™ precast Bis-Tris 4–12% gels were used. Gels were run using NuPAGE™ MES SDS Running Buffer and NuPAGE™ Antioxidant.

For one-dimensional electrophoresis 1, 5, 10, 20, 25, and 30 µg of AMG protein extract from both male and female unfed insects were analyzed. A uniform load of 20 µg of protein per lane was determined to be optimal because it produced the highest number of bands with the lowest amount of sample applied, providing the best resolution.

Precision Plus Protein Standards All Blue (Bio-Rad, Hercules, CA, USA) were used as molecular weight references. Gels were stained with 0.2% Coomassie Brilliant Blue R-350 (GE Healthcare Bio-Sciences AB, Uppsala, Sweden). Other gels were transferred for protein immunodetection.

### 2.5. Two-Dimensional Electrophoresis

(a) Isoelectric focusing

Proteomic maps were obtained by isoelectric focusing followed by two-dimensional electrophoresis based on previous methodologies used to characterize actin and tubulin isoforms in *Taenia solium, T. crassiceps* cysticerci, and *T. cruzi*, as well as in the intestine of *M. pallidipennis* nymphs and adults [40]. Eighty micrograms of each protein extract were analyzed using 3–10 pH, 7 cm immobilized gradient strips (Bio-Rad). Strips were hydrated for 16 h at 20 °C using the Protean i12 IEF Isoelectric Focusing Cell (Bio-Rad) in a buffer containing 7 M urea (GE Healthcare Life Sciences, Marlborough, MA, USA), 2 M thiourea (GE Healthcare Life Sciences), 4% CHAPS (Sigma Life Science, Oakville, ON, Canada), 0.002% bromophenol blue, 60 mM dithiothreitol, and 2% immobilized pH gradient buffer pH 3–10 (Cytiva 17600087). Isoelectric focusing was performed using the 3–10 G program in four sequential steps: (1) 250 V for 15 min with a rapid voltage ramp; (2) 4000 V for 1 h with a gradual voltage ramp; (3) 4000 V with a rapid ramp until reaching a total of 15,000 Vh; (4) 500 V hold until all strips reached 15,000 Vh. The current was limited to 50 µA per strip throughout the run. After completion of the isoelectric focusing, strips were stored at −80 °C until the next electrophoretic run.

(b) Two-dimensional electrophoresis

Isoelectric focusing strips were incubated in an equilibration buffer containing 1.5 M Tris-base (pH 8.8), 7 M urea, 30% glycerol, 2% SDS, and 0.002% bromophenol blue, along with 64.8 mM dithiothreitol and 153.1 mM iodoacetamide. Then, strips were placed on a NuPAGE 4–12% Bis-Tris ZOOM Gel (1.0 mm × immobilized pH gradient well) (Invitrogen) and fixed with 0.5% agarose (Bio-Rad). Electrophoresis was performed as described above for one-dimensional electrophoresis. Proteomic maps were stained or transferred.

### 2.6. Western Blotting

One- and two-dimensional electrophoresis gels were transferred to polyvinylidene fluoride membranes (Immobilon-P, Merck Millipore, Burlington, MA, USA) using a Trans-Blot Turbo system (Bio-Rad) at 15 V for 30 min with transfer buffer (Invitrogen) containing antioxidants. Membranes were stored at −20 °C until antibody detection.

Three antibodies were used for protein detection: anti-alpha-tubulin (mouse monoclonal, clone DM1A, 1:1000); anti-beta-actin (rabbit monoclonal, clone RM112, 1:1000); and anti-heat shock protein HSP70 (mouse monoclonal, clone BRM-22, 1:1000). All were purchased from Sigma-Aldrich (Burlington, MA, USA).

Membranes were incubated in a blocking solution containing the primary antibody for 1 h, followed by species-specific secondary antibodies for 30 min. Mouse anti-IgG (1:1000) was used for anti-tubulin and anti-heat shock protein HSP70, and rabbit anti-IgG (1:2000) was used for anti-actin detection. This detection was performed using 3,3′-diaminobenzidine and hydrogen peroxide. Three antibody detections of separations of the same protein pool were performed.

### 2.7. Image Capture and Processing

Images of one- and two-dimensional electrophoresis gels and immunochemical reactions were recorded and processed with a Gel Doc XR documentation system (Bio-Rad). Molecular masses and number of protein bands were estimated using Quantity One v.4.6 software (Bio-Rad). Master proteomic maps were obtained by processing proteomic maps using PD-Quest v.7.4 software (Bio-Rad).

### 2.8. Results Analysis

The molecular masses of protein bands were determined using Quantity One software, based on pre-stained molecular weight standards. The experimental error was estimated within ±20% of the theoretical values established for each antibody [42].

Western blot detection levels were quantified by measuring immunorecognition density (counts/mm^2^) using the Quantity One software. Total density in one-dimensional electrophoresis and proteomic maps was calculated as the sum of all immunoreactive signals detected with the same antibody. Ratios in arbitrary units were calculated by dividing the highest value obtained for each antibody by the lowest detection level. Detection levels were compared between unfed and fed insects within the same region and sex. These ratios indicate how much the detection level in one experimental group exceeded that of the other. For graphical representation, ratios were plotted using a logarithmic scale (base 10) on the *Y*-axis to facilitate comparison among experimental groups.

Protein distribution in proteomic maps was estimated manually from the developed blots. First, the start and end points of each strip were identified. Then, the distance between these points and the antibody immunodetections was measured. Based on these measurements, the pH range corresponding to each immunodetection was calculated. Detection regions were virtually segmented along the length and width of the blots according to the distribution of immunoreactive signals. Finally, all sections were organized into a mosaic based on molecular weight, pH, experimental group, antibody, intestinal region, and sex.

## 3. Results

### 3.1. Macroscopic Characteristics of Intestinal Contents

The macroscopic examination of the intestinal tissues revealed clear differences between feeding conditions and anatomical compartments. In fed insects, the AMG contained a reddish material consistent with remnants of ingested blood, whereas the RE showed darker, brownish contents, likely corresponding to processed content. In contrast, in unfed insects both tissues appeared largely devoid of visible contents: the AMG presented a collapsed, sac-like morphology, and the RE appeared dense and opaque, reflecting the tissue itself rather than luminal material. All intestinal tissues were thoroughly washed prior to analysis to minimize residual contents, including ingested blood and associated microbiota.

### 3.2. Protein Profiles of the AMG and RE Tissues of Triatoma pallidipennis

**Figure 1:**
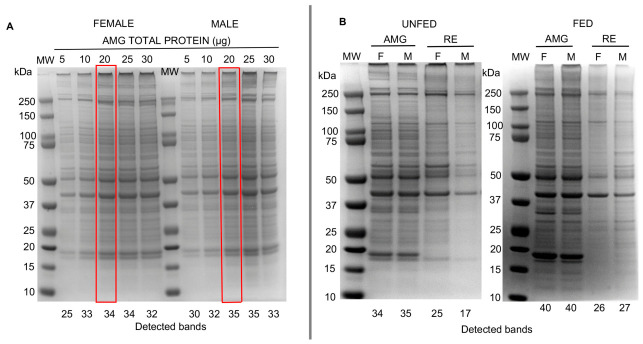
Protein profiles of the AMG and RE tissues of *Triatoma pallidipennis* analyzed using one-dimensional electrophoresis under reducing conditions using precast NuPAGE Bis-Tris 4–12% gels. (**A**) Protein profiles of AMG tissues from unfed females and males at increasing protein loads (5–30 µg). Red boxes highlight the selected optimal protein load (20 µg) used in subsequent analyses. (**B**) Protein profiles of AMG and RE tissues from unfed and fed insects, females (F) and males (M), using 20 µg of protein per lane. Gels were stained with Coomassie Brilliant Blue. Numbers below each lane indicate the total number of protein bands detected by the documentation system. Molecular weight (MW) is indicated in kilodaltons (kDa).

The electrophoretic migration of protein extracts revealed consistent banding patterns across samples. A greater number of protein bands were detected in tissues of fed insects compared to unfed insects. However, electrophoretic separation patterns of the same gut tissue from females and males showed similar distributions and abundances (Figure 1B).

Overall, the AMG tissues exhibited a higher number of protein bands than the RE tissues, particularly under fed conditions, indicating greater protein diversity in this tissue. Differences in band intensity and distribution were also observed between feeding conditions and sexes (Figure 1).

Specifically, 34 and 35 protein bands were detected by the documentation system in the AMG tissues of unfed females and males, respectively, compared to 25 and 17 bands in the RE tissues of unfed females and males, and 26 and 27 bands in the RE tissues of fed females and males.

Protein bands in the AMG tissues also showed higher abundances than those in the RE tissues, particularly at molecular masses of 300, 250, 58, 50, 41, 18, and 17 kDa, which were prominent in both tissues of fed and tissues of unfed insects (Figure 1B).

In the RE tissues, 25 protein bands were detected in unfed females compared to 26 in fed females, whereas in males a marked reduction was observed in tissues of unfed insects (17 bands). Additionally, among tissues of unfed insects, an eight-band difference was observed between females and males (25 vs. 17 bands).

### 3.3. Western Blot from One-Dimensional Electrophoresis

**Figure 2:**
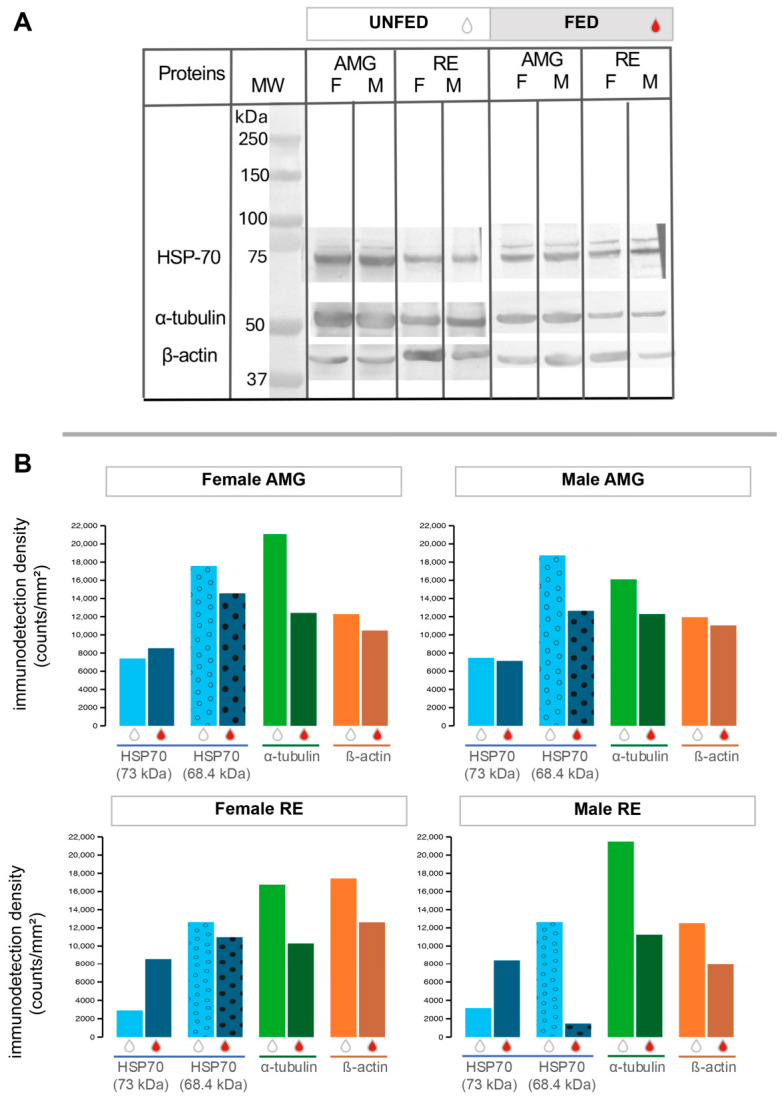
Western blot analysis of cytoskeletal proteins and HSP70 in AMG and RE tissues of *Triatoma pallidipennis*. (**A**) Immunodetection of HSP70 (73 and 68.4 kDa), α-tubulin (51 kDa), and β-actin (39.4 kDa) in tissues of unfed and fed insects, females (F) and males (M), using 20 µg of protein per lane. Molecular weight markers are indicated in kilodaltons (kDa). (**B**) Quantification of immunodetection density (counts/mm^2^) of HSP70 isoforms (73 kDa and 68.4 kDa), α-tubulin, and β-actin in tissues of females and males under unfed and fed conditions. Bars are color-coded by protein. Feeding condition is indicated by drop symbols (white drops = unfed, red drops = fed). The two HSP70 isoforms are distinguished by bar patterns: dotted bars for the 68.4 kDa isoform and solid bars for the 73 kDa isoform. Data are shown separately for each intestinal tissue (AMG and RE) and sex.

HSP70s was recognized at 73 and 68.4 kDa in one-dimensional electrophoretic separations in tissues of unfed and tissues of fed insects (Figure 2A). Immunodetection at 68.4 kDa was 1.3–2.2 times higher than at 73 kDa within the same intestinal tissue and sex. Detection densities at 73 kDa were similar across intestinal tissues and sexes. However, at 68.4 kDa, higher detection densities were observed in the AMG tissues of unfed insects, whereas this pattern was reversed in the RE tissues of fed insects for both sexes (Figure 2B).

The molecular masses detected by the three antibodies differ from theoretical values (Table 1); however, these values fall within the expected experimental range for proteomic analyses of this type [42]. The two cytoskeletal proteins showed a higher immunodetection density in tissues of unfed insects. Specifically, α-tubulin was detected at 51 kDa and β-actin at 39.4 kDa (Figure 2A). Both proteins were 1.3–1.85 times more abundant in tissues of unfed insects compared to tissues of fed insects (Figure 2B).

### 3.4. Proteomic Maps of the AMG and RE Tissues of Triatoma pallidipennis

**Figure 3:**
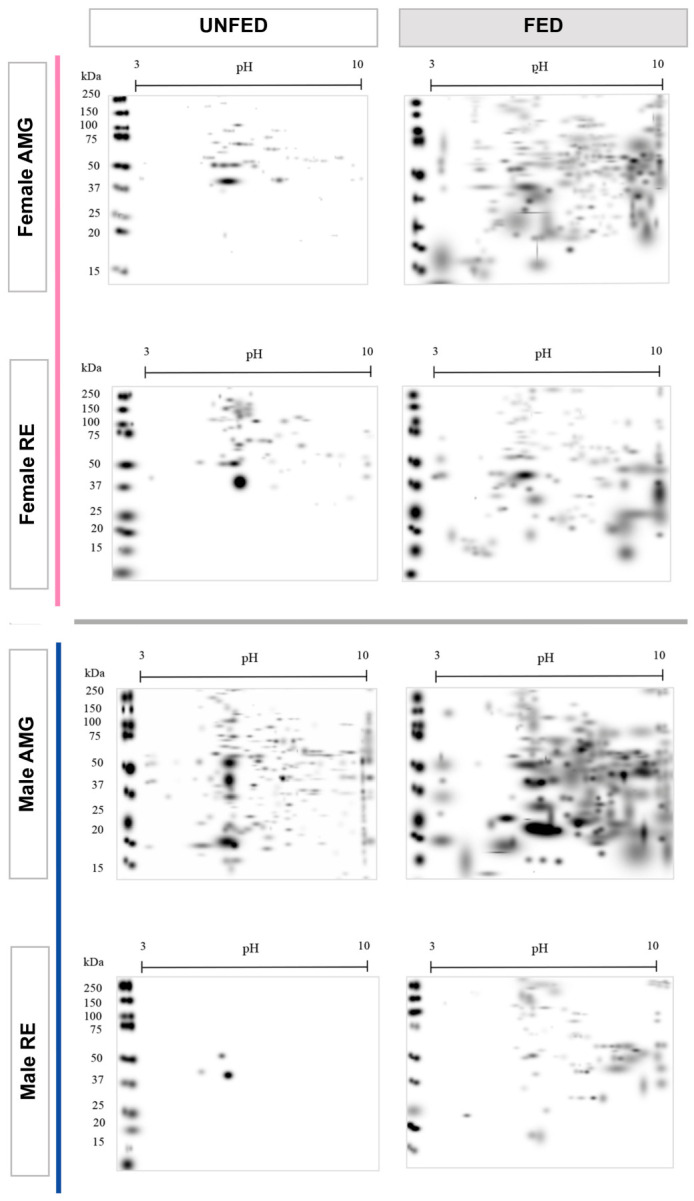
Master proteome maps of AMG and RE tissues of *Triatoma pallidipennis*. Two-dimensional electrophoresis (80 µg protein) of tissues of unfed and fed insects, females and males. Proteins were separated using immobilized pH gradient strips (pH 3–10, 7 cm) and 4–12% Bis-Tris gels, followed by Coomassie Brilliant Blue staining. Panels are organized by feeding condition (columns: unfed and fed) and by intestinal tissue and sex (rows: Female AMG, Female RE, Male AMG, Male RE). Pink frames indicate female tissues, and blue frames indicate male tissues. Molecular weight markers (kDa) and pH ranges are indicated for each gel.

Master proteomic maps revealed distinct patterns among experimental groups, intestinal tissues, and sexes. Overall protein abundance was higher in tissues of fed insects than in tissues of unfed insects (Figure 3).

### 3.5. Western Blot from Proteomic Maps

**Figure 4:**
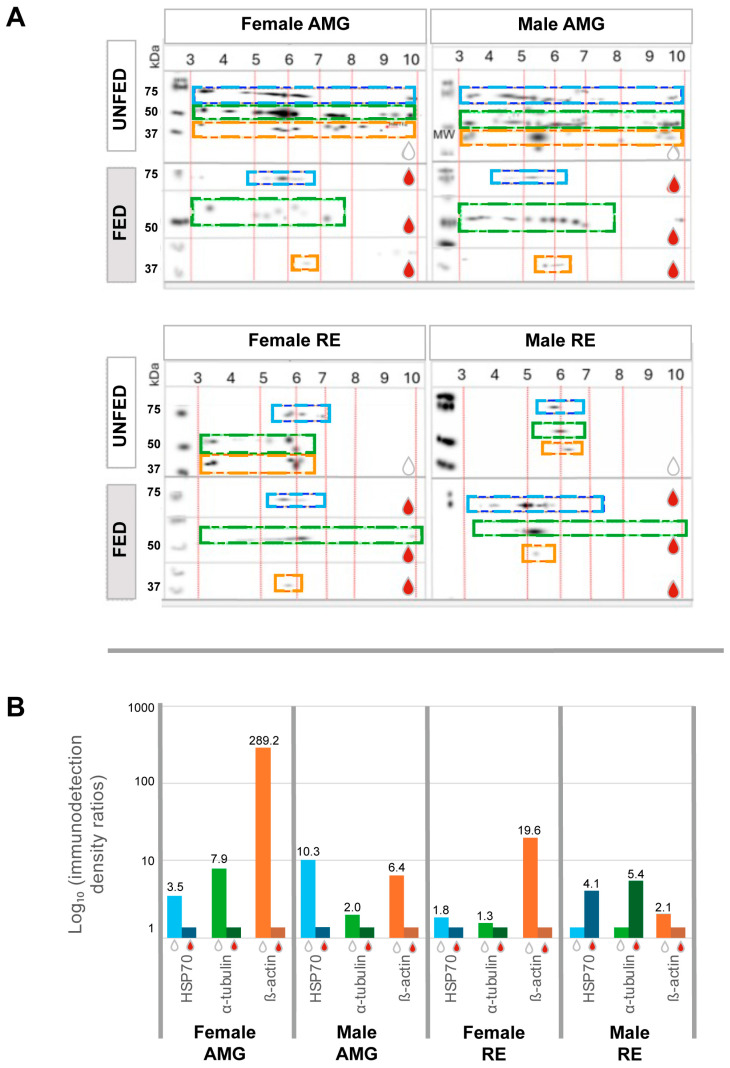
Western blot analysis from proteomic maps showing the distribution and relative abundance of HSP70, α-tubulin, and β-actin in *Triatoma pallidipennis* (**A**) Localization of immunodetected proteins in the AMG and RE tissues of unfed and fed insects, females and males. Immunoreactive signals were mapped according to molecular weight and isoelectric point (pH) following two-dimensional electrophoresis. Colored dashed boxes indicate the regions where each protein was detected: HSP70 (blue), α-tubulin (green), and β-actin (orange). White drops indicate unfed insects, and red drops indicate fed insects. (**B**) Quantitative analysis of immunodetected proteins in the AMG and RE of females and males under unfed and fed conditions. Bars represent ratios derived from immunodetection density (counts/mm^2^). Ratios were calculated as the quotient between the highest and lowest detection values for each antibody. The *Y*-axis is displayed on a logarithmic scale (log_10_) to facilitate comparison among experimental groups.

The AMG tissues of unfed insects of both sexes exhibited high immunodetection densities for all three proteins. These proteins were distributed across the pH gradient with distinct abundance patterns (Figure 4A,B).

Protein spots in the intestinal tissues of fed insects were well defined, whereas those in tissues of unfed insects appeared more diffuse. HSP70 was detected in the AMG tissues within a pH range of 4.5–6.5 in both sexes. Tubulin was detected between pH 3 and 7.5, while actin was detected between pH 5.8 and 6.3. In the RE tissues, protein detection patterns varied between sexes. In females, HSP70 was detected between pH 5.5 and 6.5, whereas in males it ranged from pH 3.5 to 6. Female α-tubulin was detected between pH 4 and 6, and male α-tubulin between pH 4.8 and 5.5. β-actin was detected at pH 5.5–5.8 in females and at pH 5.3 in males.

Overall, protein abundance was lower in the RE tissues than in the AMG tissues. In males, higher immunodetection was observed in tissues of fed insects compared to tissues of unfed insects, whereas in females a similar distribution was observed between conditions, with slightly higher abundances in tissues of unfed insects (Figure 4A,B).

## 4. Discussion

Food availability is a major determinant of physiological and structural adjustments in hematophagous insects. In this study, protein patterns differed between intestinal tissues and feeding conditions, with the AMG showing greater protein abundance than the RE, particularly in tissues of fed insects. This is consistent with the role of the AMG as the primary site for blood storage and initial digestion, where the presence of food promotes higher metabolic and digestive activity, as reported in triatomines [8,20].

In contrast, the RE tissues exhibited lower variation in protein abundance between tissues of fed and tissues of unfed insects. This relative stability is consistent with its role in water reabsorption, ion balance, and excretion, processes that must be maintained regardless of feeding status [27]. The reduced variability observed in this tissue suggests that its physiological functions are less influenced by short-term nutritional changes compared to the AMG tissues.

A notable finding of this study is the higher abundance of α-tubulin, β-actin, and HSP70 in the AMG tissues of unfed insects. Rather than indicating increased protein synthesis, this pattern likely reflects adjustments associated with cellular maintenance under nutritional stress. Cytoskeletal proteins such as actin and tubulin are essential for maintaining epithelial structure, intracellular transport, and membrane organization [19,43]. Under food deprivation, these functions may become particularly important for preserving tissue integrity and enabling a rapid response upon refeeding.

The increased abundance of these proteins may also be associated with cellular processes involved in structural remodeling during nutritional stress. For example, cytoskeletal components have been implicated in membrane dynamics and intracellular trafficking in various physiological contexts [20]. Although autophagy was not directly evaluated in this study, previous reports in insects indicate that actin and tubulin participate in cellular reorganization under nutrient-limited conditions [44,45,46,47,48]. Therefore, the patterns observed here are consistent with adaptive responses aimed at maintaining cellular homeostasis during fasting.

Similarly, the higher abundance of HSP70 in tissues of unfed insects suggests a role in cellular protection under stress conditions. HSP70 is a molecular chaperone involved in protein folding, stabilization, and prevention of aggregation, and its induction has been associated with physiological stress, including nutritional restriction in insects [20,26,49]. In this context, the observed increase in HSP70 may contribute to maintaining protein functionality during periods of reduced metabolic activity.

The differences observed between intestinal tissues further support the idea of tissue-specific functional specialization. While the AMG tissues appear to undergo dynamic adjustments in response to feeding conditions, the RE tissues maintain a more stable protein profile, consistent with their continuous role in osmoregulation and content processing [26,49]. These findings highlight the importance of considering both anatomical tissue and nutritional status when interpreting proteomic patterns in triatomines.

An additional aspect to consider is the marked difference in protein patterns despite loading identical protein amounts, particularly in the RE tissues of unfed males and the AMG tissues of unfed females. These differences may reflect variations in protein composition and distribution associated with tissue-specific physiological roles under fasting conditions. Alternatively, this pattern may also be influenced by the electrophoretic behavior of low-molecular-weight proteins, which can migrate faster and, under certain running conditions, approach or slightly surpass the electrophoretic front. This effect may be more evident when multiple gels are processed simultaneously, potentially leading to minor variations in migration distances. Therefore, both biological and technical factors should be considered when interpreting these patterns.

Finally, no major differences were observed between females and males, suggesting that the patterns described are primarily driven by feeding condition and intestinal tissue rather than sex. Therefore, the physiological responses to food deprivation appear to be broadly conserved in both sexes of *Triatoma pallidipennis*.

Overall, according to the presented results, food deprivation does not simply reduce protein abundance in intestinal tissues of *Triatoma pallidipennis*, but is associated with tissue-specific adjustments in proteins involved in structural organization and stress response. These changes likely contribute to maintaining intestinal integrity and functional readiness during prolonged periods without feeding.

## 5. Conclusions

Food deprivation differentially modulates protein abundance in intestinal tissues of *Triatoma pallidipennis*. The AMG tissues exhibit the most evident changes associated with nutritional condition, whereas the RE tissues show comparatively minor variation. The cytoskeletal proteins α-tubulin and β-actin, together with HSP70, are consistently more abundant in tissues of unfed insects, and this pattern is independent of sex, suggesting that their abundance depends primarily on feeding status and intestinal tissue.

Overall, these findings define a tissue-specific protein abundance profile associated with fasting and contribute to the characterization of intestinal physiology in a vector of *Trypanosoma cruzi*, identifying proteins responsive to nutritional condition.

## Figures and Tables

**Table 1:** Comparison of theoretical and experimental molecular masses (kDa) of detected proteins. Values correspond to the mean ± standard deviation in four separations.

Protein	Molecular Masses (kDa)		
	Theoretical	Coomassie Blue	Antibody Detection
HSP70	~70	71.64 ± 14.33	73.0 ± 14.6
HSP70	~70	68.40 ± 13.68	68.4 ± 13.7
α-tubulin	50	49.76 ± 9.95	51.0 ± 10.2
β-actin	~45	40.40 ± 8.08	39.4 ± 7.88

## Data Availability

All data generated in this study are available within this article.
